# Echoes of the Convention

**Published:** 1895-10

**Authors:** 


### ECHOES OF THE CONVENTION.

Iowa may well be proud of the largest national veterinary convention ever held in the West.

No convention ever covered more work in three days than was disposed of at Des Moines.

There were too many familiar convention faces absent at Des Moines.

Buffalo will be a sure winner for 1896. She seems to have the field clear.

Many were the inquiries for Treasurer Robertson and his eagerly-nursed boomlet for California for 1897.

Where was California's delegate to the Des Moines convention?

Minnesota may feel more than elated at the splendid delegation she sent to the convention, many of whom joined the Association ranks during the session.

Ohio and New York joined in the selection of a hotel unknown to but few in Des Moines. They had so much company, large and small, that one night of eternal vigilance led them to seek other pastures.

Pennsylvania sent a fair delegation only, but they were strong Association men, and their faces are always to be seen East or West. Rayner, Foelker, Hoskins, Pearson, and Harger have strong Association ties.

For the first time in many years Maryland was without a representative.

Kansas and Missouri had strong delegations of earnest men.

There were some members from Illinois whom we are always glad to see at these conventions.

Michigan was represented by our ever-faithful member, Brenton, of Detroit. Where are our other colleagues?

The Southern States sent two of her most earnest veterinary representatives to aid in the consideration of the many problems for discussion.

By unanimous vote the President will be in the future intrusted with power to allot the time for committee reports and papers. This will prove a wise step, when judiciously used. The overloaded programme at Des Moines, following that of Philadelphia and Chicago, forced the necessity.

California will not be allowed a free field for 1897. Minnesota and Missouri will be strong factors then.

It is dangerous to prepare firearms for others to shoot. One of the State Association reports proved to be loaded.

California's delegate failed to materialize. Will her State Association rise and explain?

California threatens to be known as "Robertson's lost cause."

Several important papers and State Secretaries' reports were read only by title.

"Where, oh where, was Treasurer Robertson?" became a loud refrain at Des Moines.

Tuberculosis in its now every-day import to every local veterinarian should have a whole day for consideration in 1896.

Newly elected officers of U. S. V. M. A.: W. Horace Hoskins, President, 3452 Ludlow Street, Philadelphia, Pa. F. H Osgood, 50 Village Street, Boston, Mass.; C. C. Lyford, Minneapolis, Minn.; and R. H. Harrison, Atchison, Kan., Vice-Presidents. S. Stewart, Secretary, 7½ St. James Street, Kansas City, Kan. James L. Robertson, Treasurer, 409 Ninth Avenue, New York City.

---

Dr. M. E. Conard, of West Grove, Pa., recently addressed the Farmers' Institute, at Lititz, of the same State, on the "Diseases of Dairy Cows." Secretary Edge, of the State Board of Agriculture, spoke at some length on the prevalence of tuberculosis among dairy herds.

The Veterinary Medical Association of New Jersey will hold their thirty-fifth regular meeting on Thursday, October 10, 1895, at 10 A.M., at 842 Broad street, Newark. The Secretary announces the fact of questions of vital importance to be considered and urges a full attendance, and we join him in saying that there should be no State with a stronger veterinary organization than New Jersey, for she has within her borders many of the strongest individual members of the profession in the Middle States.

